# Vitamin B6: A new approach to lowering anxiety, and depression?

**DOI:** 10.1016/j.amsu.2022.104663

**Published:** 2022-09-15

**Authors:** Duaa Durrani, Rahma Idrees, Hiba Idrees, Aayat Ellahi

**Affiliations:** a Department of Internal Medicine, Dow University of Health Sciences, Karachi, Pakistan; b Department of Internal Medicine, Karachi Medical and Dental College, Karachi, Pakistan; c Department of Internal Medicine, Jinnah Sindh Medical University, Karachi, Pakistan

**Keywords:** Vitamin B6, Anxiety, Depression, Stress, Vitamin B12, Pyridoxine

Dear Editor,

Anxiety and depression are common medical illnesses that cause adverse effects on the body, mood, and thoughts. Anxiety and mood disorders are the most pressing public health issues in today's world, affecting 14% of the global population [1]. Around 480 million people worldwide are currently depressed, and approximately a quarter also suffering from anxiety [2]. Such conditions are currently treated with cognitive and dialectical behavioral therapy, as well as medications such as benzodiazepines and buspirone.

It has recently been discovered that taking high doses of vitamin B6 supplements significantly reduces feelings of stress, anxiety, and depression. A study conducted by a group of researchers at the University of Reading, issued in the renowned journal, Human Psychopharmacology: Clinical and Experimental, found that after monitoring the effects of an elevated dose of vitamin B6 on young adults for more than a month, the participants reported feeling less anxious [3]. The study adds to the growing body of evidence supporting the use of such supplements to improve cognitive function, treat mood disorders, and hence boost mental well-being.

The researchers first gathered 478 participants with self-reported depression and/or anxiety who were randomly assigned to either Vitamin B6, or Vitamin B12, or a placebo. The researchers wanted to know how vitamins B6 and B12 influence the way gamma-aminobutyric acid (GABA) is used [3]. Vitamin B6 (Pyridoxine) has a significant and selective modulatory impact on central serotonin and GABA production [4]. GABA is a chemical messenger and inhibitory neurotransmitter found in the brain. It helps calm the nervous system by blocking certain impulses between nerve cells, immediately slowing down brain activity. This, in turn, has a calming effect that can help relieve stress, anxiety, and fear. According to the study's findings, the B12 group experienced a minor improvement in anxiety and depression when compared to the placebo group. Above all, Vitamin B6 produced a statistically significant difference. Higher GABA levels were found in participants who took B6 supplements, as confirmed by the Mood and Feelings Questionnaire (MFQ) and Screen for Adult Anxiety Related Disorders (SCAARED) tests at the end of the trial, which screened participants for anxiety and depressive symptoms before and after the vitamin or placebo regimen [3]. A large cross-sectional study discovered that the average intake of vitamin B6 (mg/day) in anxious and depressed people was significantly lower than in healthy participants [5]. Another eight-week Phase IV randomized controlled study found that the combination of magnesium supplements and Vitamin B6 resulted in greater physical activity in everyday life and a significant reduction in stress in healthy individuals with severe stress and anxiety and low magnesemia [6].

Therefore, taking the aforementioned study results into consideration, it can be concluded that vitamin B6 actively helps in relieving symptoms that accompany depression and anxiety, making it an important supplement to be added to our daily lives. Another important consideration is that, while benzodiazepines are effective in treating anxiety and other similar conditions, they also have a number of side effects, including but not limited to dependence, rebound anxiety, memory impairment, and discontinuation syndrome [7]. Whereas, assuming that B6 toxicity is of little significance, it is vital that it be included to everyone's diet. It's important to remember that the research is still in its early stages and that Vitamin B6's effect on anxiety in the study was relatively trivial, compared to what might be expected from the drug. People may prefer nutrition-based interventions in the future because they have fewer negative side effects than drugs. To make this a viable option, more research is needed to identify other nutrition-based interventions that improve mental well-being. In the future, different dietary interventions could be combined to produce better results. Using Vitamin B6 supplements in conjunction with talking therapies such as Cognitive Behavioral Therapy could be one option for increasing their effectiveness.

## Please state any sources of funding for your research

No funding was acquired for this paper.

## Ethical Approval

This paper did not involve patients, therefore no ethical approval was required.

## Consent

This study was not done on patients or volunteers, therefore no written consent was required.

## Author contribution

Duaa Durrani: conception of the study, drafting of the work, final approval and agreeing to the accuracy of the work.

Rahma Idrees: conception of the study, drafting of the work, final approval and agreeing to the accuracy of the work.

Hiba Idrees: conception of the study, drafting of the work, final approval and agreeing to the accuracy of the work

Aayat Ellahi: conception of the study, drafting of the work, final approval and agreeing to the accuracy of the work.

## Registration of Research Studies

Name of the registry: Not Applicable

Unique Identifying number or registration ID: Not Applicable

Hyperlink to your specific registration (must be publicly accessible and will be checked): Not applicable

## Guarantor

Duaa Durrani, Rahma Idrees, Hiba Idrees, Aayat Ellahi.

## Declaration of competing interest

The authors declare that there is no conflict of interest.
